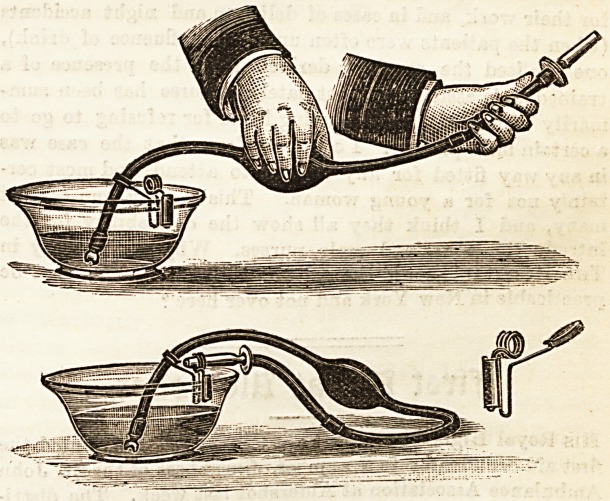# The Hospital Nursing Supplement

**Published:** 1895-12-28

**Authors:** 


### "The Hospital" Nursing Mirror.

Being the Extra Nursing Supplement of "The Hospital" Newspaper.

[Contributions for this Supplement should be addressed to the Editor, The Hospital, 428, Strand, London, W.C., and should have the word "Nursing" plainly written in left-hand top corner of the envelope.]

### News from the Nursing World.

### CHRISTMAS GREETING.

To all our readers we send cordial greetings for a Happy Christmas and a Glad New Year, and we hope that through their efforts to make the season pleasant for the sick under their care, they have also ensured enjoyment for themselves.

### BOYS AT MIDDLESEX HOSPITAL.

The ward for boys at Middlesex Hospital is a very cheerful place, with its numerous windows, and bright fire, and polished floor. The visitor is tempted to linger and listen to the quaint sayings of little people, who are so evidently on good terms with themselves and each other. Whilst one boy appeals to sister for confirmation of his statement that he cannot sing just now because his neck has been operated on, another small person is pointed out by his neighbours as an accomplished vocalist, and his sweet voice is soon heard trilling out many verses of a song "learnt me by my big brother; he belongs to a choir, you know." Christmas is anticipated with pleasant confidence by these patients, who are shrewdly alive to their own popularity with all the officials, from the matron downwards.

### ENTERTAINMENTS AT THE ROYAL FREE HOSPITAL.

Christmas was made pleasant in various ways at the Royal Free Hospital, a concert being arranged by the chaplain, festive ward teas by the nursing staff, and two excellent entertainments in an unused ward by the women students. The performances included some plantation songs charmingly rendered by the Pierrot Troupe, a most amusing edition of "Mrs. Jarley's Waxworks," and two one-act plays. The enthusiasm displayed by the audience was sufficient proof of their enjoyment of the really excellent acting, and the patients are not likely soon to forget the entertainments which no pains had been spared to make such a brilliant success.

### AN IMPORTANT CONFERENCE.

The chairman of the Parliamentary Bills Committee, British Medical Association, proposes to call a conference in January of the various nursing bodies, with the object of asking their opinion as to the expediency of obtaining an Act of Parliament providing for the registration and education of surgical, medical, and obstetric nurses. On the motion of Dr. Bedford Fenwick, seconded by Mr. John Brown, a resolution was passed at the last annual meeting of the British Medical Association recommending that such an Act should be passed as soon as possible. The Council of the Association has referred the matter to the Parliamentary Bills Committee, of which Mr. Ernest Hart is chairman, and the conference about to be held will give an opportunity for those who have made the education and training of nurses their special study, to give their views with respect to State registration. A decision on this matter is of supreme importance to the whole body of nurses, and it is therefore desirable that the conference should be as representative as possible. Attendance at this preliminary gathering will in no way pledge anyone present to countenance any movement of which they do not cordially approve.

### NEW HOSPITAL FOR WOMEN.

Carol singing and other seasonable entertainments have been arranged at this pretty little hospital. The women students, who gave so much pleasure to the patients at the Royal Free Hospital by means of the Pierrot Troupe and "A Dress Rehearsal," repeated these performances on the following evening at the New Hospital for Women in Euston Road.

### NEWTOWNARDS NURSING SOCIETY.

The annual meeting of the Newtownards Nursing Society was held last week, and the financial condition of the society was shown to be most satisfactory. The Needlework Guild has proved a valuable adjunct, and much local interest and support have been given to the society, which owed its origin to the kind efforts of Lady Londonderry, the president.

### A NURSE FOR CHARD WORKHOUSE.

An instance of the primitive ideas which still prevail in some districts with regard to sick nursing was recently given at a meeting of the Chard Guardians. It appears from the local press report that the Local Government Board objects to sanction the appointment, as nurse at Chard Workhouse, of a woman who has had no previous experience of the duties required. The Guardians seem unable to realise the preparatory training required to make a competent nurse, for after disparaging the course taken by the Local Government Board, they agreed to appeal to that body to confirm the appointment they desire for a period of six months. Perhaps they imagine the Local Government Board might eventually be persuaded to consider six months' service in the wards as adequate training for the candidate in question.

### DISTRICT NURSING FOR HUNTINGDON AND GODMANCHESTER.

At a recent public meeting at the Court Hall, where the Mayor of Godmanchester was in the chair, it was proposed, seconded, and carried that Godmanchester should share with Huntingdon in the maintenance of a trained district nurse for the poor of the neighbourhood. Archdeacon Vesey attended the meeting on behalf of Huntingdon. A committee was appointed, and a party of ladies empowered to confer with the promoters of the scheme at Huntingdon.

### GLASGOW NURSES' CO-OPERATION.

The third annual report of the Glasgow and West of Scotland Co-operation for Trained Nurses was laid before the members at the general meeting. There are now 46 nurses on the staff, and they have been well employed during the past year. The committee hope that the association will become selfsupporting at an early date, and to further this end 10 per cent. is charged on the earnings of the members for working expenses, instead of the 7½ per cent. which was tried at first. It is not considered desirable to have a larger staff, and the present staff is not sufficiently numerous to be entirely self-supporting. The rules appear to be good and workable, and there is a nurses' residential club, also a general sitting-room, of which nurses who do not live in the home can have the use on payment of an annual subscription. Miss Rough, 18, Sardinia Terrace, Hillhead, Glasgow, is the lady superintendent.

### THE ARBROATH ASSOCIATION.

The Arbroath District Nursing Association has just published its fourth annual report, which contains an excellent record of work. The association is affiliated with the Queen's Jubilee Institute, and the work of the nurse appears to be very highly esteemed by her patients. She has attended 408 cases in the course of the year.

### NEW ROOMS FOR PARISIAN NURSES.

The need for better accommodation for the nursing staff in Paris hospitals is receiving consideration, and the Municipal Council has voted a sum of money for the construction of proper quarters in connection with the Necker Hospital. So much has been done to improve this hospital, that the contemplated nurses' home will probably fulfil all modern requirements. The Necker Hospital is fortunate in the possession of extensive and beautifully-kept gardens. The Municipal Council has also voted for the provision of nurses' quarters at the Children's Hospital, whose six hundred patients are accommodated in a number of detached blocks of buildings.

### WALLSEND NURSING ASSOCIATION.

The first annual report of the Wallsend Nursing Association shows that the work of Nurse Rudd has proved most acceptable in the district. One hundred and ten cases have been visited, and 1,178 visits paid. The committee are desirous of finding a probationer to work under the district nurse, and they acknowledge most gratefully the useful gifts received by the association,

### PRIVATE NURSES IN SAN FRANCISCO.

A correspondent, writing to our contemporary, *The Trained Nurse*, gives a deplorable account of the class of nurses registered at a San Francisco agency. She says that there are over three hundred names on the books, some of which belong to women who have never been inside a hospital.

### A PLEA FROM IRELAND.

An urgent appeal for assistance has reached us from Sister M. Dominico, of the Mercy Hospital, Gort, Galway, who begs that the poor under her care may have a share of Christmas comforts. We are forwarding to her a parcel of warm clothing from the needlework competition.

### THE AVERAGE VALUE OF CERTIFICATES.

It is apparent that the question of quality as well as quantity in the matter of training is beginning to present itself to our sister nurses in America. Our correspondent told us last week that there is general discontent felt because of the misleading importance attached indiscriminately to certificates. This bears out our own argument that two years (the minimum period which can be approved) passed in the well-ordered training school of a general hospital or large infirmary will develop a far more useful kind of nurse than three or four years passed in a very small general or special hospital. Many English nurses share the disgust of their American sisters at the lack of discrimination betrayed by the public as to the relative value of certificates. With regard to the most complete kind of training (which is a question often asked by nurses), we are of opinion that two or three years in a general hospital, followed by a certain amount of fever and maternity training, would form far better preparation for future work than three or four spent exclusively in one of the many small institutions which now represent themselves as giving adequate training to probationers.

### INTERMITTENT TRAINING.

Nurses are inclined to think it hard that short terms of hospital training, taken perhaps at long intervals, "don't count." They say it is not fair that the same amount of time if spent consecutively in hospital should confer a higher position on a fellow nurse. But surely the art of nursing must be acquired as thoroughly as that of teaching, and no one expects to gain a certificate by intermittent attendance at school. It is, no doubt, hard in certain individual cases when regular training has been unavoidably prevented, but it is useless to make a misfortune into a grievance. It is, to our thinking, far worse when a probationer who has patiently worked for three years in a small institution or special hospital finds herself in possession of a certificate of no value whatever in the nursing world.

### DISTRICT NURSES IN LANCASHIRE.

The Sick Poor and Private Nursing Institution, Chorlton-on-Medlock, has been in existence for thirty years, and numbers 41 nurses on its staff. In presiding over the annual meeting, which was recently held in the Memorial Hall, the Bishop of Manchester spoke most eloquently of the work done by the nurses, and of the obvious advantages in many cases of securing trained attendance for the sick poor in their homes.

### SHORT ITEMS.

The Nursing Guild in connection with the York centre of the St. John Ambulance Association have engaged the services of a Queen's Nurse.—An excellent concert was held in the Workmen's Hall in aid of the funds of the Radstock Sick Nursing Society.—On her resignation of the post which she has held for over eight years at Brighton Workhouse, Nurse E. J. Clark has received a most gratifying testimonial from those who lose her valuable services with much regret. —A movement to secure a trained district nurse for Elgin is receiving considerable local encouragement.—Miss Rees has been appointed head nurse at Stepney Workhouse.—A sale of work in aid of the funds of the Plymouth Homœopathic Hospital and Dispensary was recently held at the institution.—We learn with much pleasure that Mr. H. F. Cox has given chairs similar to the one described in The Hospital of November 30th, page 105, to the Charing Cross Hospital, the Hospital for Children and Women, Waterloo Bridge Road, Royal Free Hospital, National Hospital, Middlesex Hospital, and Guildford County Infirmary.

### Lectures on Nursing.

By a Superintendent of Nurses.

### II.—HEALTH, METHOD, SYMPATHY.

There are special qualifications required in an efficient nurse, namely, the power of observation, forethought, presence of mind, gentleness, accuracy, good memory, and good health. I have put this last, but I ought, perhaps to have put it first, and I will give you one or two suggestions as to the best way to preserve your health.

It is essential that you should have a good amount of fresh air, food, and sleep. Do try to get out daily, at least for an hour. Avoid the habit of lying down during your hours off duty. Remember, you are breathing more or less impure air all day long, air laden with germs of disease very different from that which you have been accustomed to breathe. It is most necessary that you should do this, or you will soon exhaust your stock of strength, become languid, have no appetite, and feel generally out of sorts. You know how difficult it is to keep travellers awake sometimes on snowy mountains. They beg to be allowed to lie down just for a little, but the guide knows it means to sleep the sleep of death; well, so it is with nurses. They feel tired and say, "Oh, I shall go and lie down," and they forget that by going into the fresh-air they will take into their lungs the life-giving element oxygen, and will come back better in every way. You can easily get on the top of a tram if too tired to walk. If you have studed physiology you know how important fresh-air is; and if you have not, let me beg you to take my advice on trust and act upon it. Unless you go out you will assuredly find your work become burdensome.

Now as to food. You may see a beautiful fire burning brightly, and say to yourself, "That fire will last for hours," but if you do not go near it for some time you will find it nearly all burnt out for want of fresh coal. So it is with our bodies. There is a constant fire burning in them, and food is, so to speak, the fuel, and you must supply them with this fuel, unless you wish the fire to burn low or go out altogether.

I would strongly urge upon you the necessity of beginning the day well in this respect. Be punctual at breakfast, and make a good meal. Remember this, if your stomach is empty you become at once susceptible to all the bad influences at work in a hospital ward, and you must not be surprised if you become sickly and feverish, or knock up with a hospital throat. Therefore do fortify yourselves by eating a good breakfast, and give yourselves time to eat it properly. I am very anxious to impress you with the fact that there will be a great strain upon your health, and it is for you to carry out such rules as will help you to preserve it; for if health gives way the spirits flag also, and your usefulness as nurses is greatly lessened. Then as to sleep. Get as much sleep at night as you can. Go to bed early so that the brain may have restored to it that which the wear and tear of the day has deprived it of.

Let your clothing be light, yet warm. Heavy clothing is very tiring. Be sure and wear flannel, for whilst you are at work you frequently get very heated, and going out of your wards you are liable to meet cold draughts in the corridors; to take cold, thus laying the foundation of perhaps serious illness. Flannel next the skin is a great protection, and a very wise precaution.

Hospital life is apt to become a very narrow life, and the work an all-absorbing one, and it is not good for nurses to get their world confined to the institution they are connected with, nor to get their views of life narrowed, and all their interest centred in the hospital of their choice. I would have you very diligent during your on-duty time, but equally diligent in refreshing mind and body in off-duty hours. Keep up your home interest. If you are only thinking of a short training this need not apply, but if you are taking up nursing as a profession do try to act on my advice. You will regret it exceedingly in after years if you let your work separate you from your home people and cause you to be out of touch with them. See your friends as often as convenient, and try in your leisure to get hold of good books. Again let me beg of you to go out daily, and when out shake off hospital thoughts, then you will come back refreshed and ready with zeal to begin work again, thankful that you are one of the world's workers. As Carlyle says, "Blessed is the man who has found his work in life." We have spoken of the need of fresh air, food, sleep, now let me speak of method—economising of labour.

A probationer suffers if her head nurse lacks method. This I found to my cost years ago, and certainly many a probationer suffers from not knowing how to economise her labour. Allow yourself time in the morning to dress with comfort, and to make your room tidy. Do not get into the slovenly way of just throwing the bed-clothes over the unmade bed, as I have known nurses do, making the outside respectable. Then, having had a good breakfast, begin your ward work with the determination not to scamp any of it, but to do thoroughly all that you have to do, however insignificant it may seem to you. Get into the way of doing things quickly, not dawdling over them nor wasting moments by chatting with your fellow-nurses, until your work is finished. Think of what you have to accomplish, and do it without fuss. At first your duties may seem like those of a housemaid. Never mind; this is a part of your training, which you will be glad some day you have gone through. Think over what you have to do, and go steadily on with it; do not leave things half done with the idea of finishing another time. If you do the latter your mind will be constantly burdened with having to recollect items of work. Get them off your mind as quickly as possible. Put things away directly you have finished with them, so that there may be no sense of disorder in your mind.

Some nurses are, however, splendidly methodical and well trained in the details of nursing, and yet their success with patients is not so great as that of others who are not so well up in the knowledge of the art. The key-note of their success is sympathy. The patient surrenders himself to doctors and nurse, and it is for both to remember their duty to their neighbour. If ever "noblesse oblige" applies to anything, it applies to your treatment of those who have placed themselves in custody in our wards. Galen laid it down as a principle that his disciples were to bring all their power, skill, and ability to bear, to prolong life and to ease suffering, and it is necessary to maintain this principle and to act upon it, letting no enthusiasm for order, punctuality, rule, or love of organization lead you to forget your first duty. Try as you look on each patient to think, "Lord, he whom Thou lovest is sick." Speak a few cheering words to new patients; show that you take an individual interest in each person. Ask about his complaint, not in a flippant way, but because you want to help him, and make him feel he has a friend near. Remember, each man is his own world so to speak.

"The paths of pain are thine; go forth
With patience, trust, and hope;
The sufferings of a sin-sick earth
Shall give thee ample scope.

"Beside the unveiled mysteries
Of life and death go, stand,
With guarded lips and reverent eyes,
And pure of heart and hand.

"So shalt thou be with power endued
From Him who went about
The Syrian hillsides, doing good
And casting demons out.

"That Good Physician liveth yet,
Thy Friend and Guide to be;
The Healer of Gennesaret
Shall walk the rounds with thee."

—*Whittier.*

### Food, with Special Relation to the Sick.

### I.—ADMINISTRATION OF NOURISHMENT.

Without treating *in extenso* of so large a subject as food in sickness, it might be useful to note one or two points that should receive special attention from a nurse, especially one engaged in private nursing. These two points are—

1. That food for the sick need not be unpalatable.
2. That it need not be monotonous.

Let us first briefly consider some of the conditions peculiar to a sick person in contradistinction to a healthy one.

The sick person has to fight against a disease which has invaded his body, and is, perhaps, causing rapid wasting of the tissues, especially if it be attended by high fever, or is one of the class known as "wasting diseases." Hence, though there be complete confinement to bed and perfect rest, nevertheless loss of flesh and consequent emaciation and weakness proceed apace, and are out of all proportion to the muscular wear of a healthy person, even of one leading a very active physical or mental life. Moreover, in health such loss can be readily replaced by food which is eaten with appetite, is enjoyed, and is easily assimilated. With the sick this is not the case, and we have to face the fourfold difficulty of (1) giving sufficient food to counteract the loss, and prevent the strength from ebbing away; (2) of a much-reduced power of assimilation; (3) of a limited choice of suitable foods; (4) of great disinclination for any kind of food. Truly, it is no easy matter to feed sick people satisfactorily, and to do so and successfully meet all these difficulties will tax to the utmost the ingenuity of both doctor and nurse.

As regards the first, *i.e.*, the amount, this must be just as much as the patient can digest, and neither more nor less. It must be remembered that so long as the food is unabsorbed by the glands in the stomach and intestine and does not enter the blood-stream, so long, for all practical purposes, it is still outside the body. The mere taking of food into the stomach is not feeding. When food is given of a kind that can be readily absorbed, then, and only then, can we be said to be really feeding the patient. Food that cannot be assimilated merely acts as an irritant to the intestine, causing great discomfort, and possibly diarrhœa and further weakening of the patient, whilst the natural disinclination for food is greatly increased.

How disease interferes with the power of assimilation, and why it should do so, is no concern of ours at the present time. We have only to deal with the fact that it does so, in a more or less marked degree, and especially in acute fevers and all diseases attended by high temperature, where, in addition to the patient's great dislike for food, some forms, and notably solids, disagree and are forbidden. In such cases all food must be fluid, because in that form, when the particles composing it are very minute and all moving freely, it can be most easily acted upon by the digestive fluids, and prepared for ready absorption by the glands.

In all acute diseases, especially when there is much fever, the patient has almost invariably a great dislike to food. The nerves of taste are blunted—there is no pleasure in even well-prepared and daintily served dishes—and often the prostration is so great that the mere effort of eating or drinking is a weariness to the patient.

This disinclination can be lessened to some extent by a little attention on the part of the attendant. The tongue is always furred in such cases. This is inevitable where only liquid food is taken, and occurs even in relative health, much more so then in sickness, when in addition the whole mouth is apt to be foul. Therefore, before giving food the mouth should be well swabbed out with pieces of lint or clean soft linen firmly wrapped round and fastened to a suitable piece of whalebone or stick or to a sponge-holder, and when firmly secured dipped in a non-poisonous antiseptic lotion—such as Condy's Fluid, two teaspoonfuls to a pint of water or a weak solution of boracic acid. The mouth and tongue may be gently rubbed with the lint or linen, and the mouth rinsed or syringed out with the lotion. Also the teeth may be rubbed in the same way, and the lint, when used, burnt, and a fresh piece taken for the next occasion. This process will be a distinct refreshment to the patient, and can easily be performed so as not to worry or weary him. Food administered after it will be ten times more enjoyed than it would have been before. When giving milk or beef-tea to a patient who is very ill, it is a good plan to give immediately afterwards a mouthful or two of fresh cold water to wash down the other, which is apt, in many cases, to hang about the mouth and fauces, entangled, as it were, in the thick mucous of the mouth. This washing down will be found a very good plan to adopt when food has to be administered frequently, as the water will prevent the taste from continuing in the mouth till the next feeding time comes round. The patient will be much more ready for the next supply, and the various administrations will not seem so frequent as they are apt to do when the taste of the food remains in the mouth in the intervals, and the patient plaintively rebels against what appears to him incessant feeding.

We think the physical comfort of a patient should be attended to in every possible way before food is administered. A person in health instinctively prefers to cool and refresh himself with a wash before partaking of a meal, and we believe some similar preparation is advisable for a sick person. The nurse should gently and rapidly sponge the face and hands of her patient with either plain water or water to which a little eau de cologne or toilet vinegar has been added, arrange pillows and bedclothes, and carry out other arrangements that will tend to make her patient cool and comfortable. Then immediately afterwards food should be presented, and we are quite sure it will be eaten with a better appetite and more enjoyed than if there had been no previou s preparation.

### "Truth" Doll and Toy Show.

On Wednesday and Thursday in last week the arena of the Albert Hall was once again filled to overflowing with a perfect children's fairy-land of dolls and toys, contributed by readers of *Truth* to the annual distribution amongst the little ones in hospitals and workhouses in London. Each year's show proves more attractive than the last, and crowds of interested visitors filled the hall during the two days of exhibition. Certainly the sight is one not to be missed. This year a slightly different plan of arrangement allowed the dolls individually to be better seen, the bulk of them being ranged in rows all around the arena, the larger ones placed on the centre stand. Instead of the bought toys being piled up as a background, this year huge packing cases completed the circle, ready filled with thousands of toys, and addressed to their various destinations. Some 22,000 these purchased toys numbered altogether. Amongst the larger dolls, one entitled "Good Luck," dressed by Miss Miller, justly drew much admiration; her white satin dress spangled over with symbols of good fortune, merry thoughts, money-spinning spiders, a black cat, &c.; round her neck a crooked sixpence; in her mouth a silver spoon, and bearing a horseshoe in her hand. "My Lady Coquette" was a charming creation, and so was another dainty lady, in filmy white and daisies, "Marguerite." "Trilby," of course, was conspicuously *en evidence*, a faithful presentment of Miss Dorothea Baird; and a group at one end of the hall showed the wife of President Cleveland, her three children, and their nurse. These were specially dressed by some ladies at Baltimore, and sent over by the proprietor of the *Baltimore American*, who has established a doll show on *Truth* lines, to which six dolls have been contributed by the editor of *Truth*, dressed by English ladies for the purpose. 22,000 crackers were as usual sent by Mr. Tom Smith, and once more 11,000 newly-minted sixpences came anonymously for children in metropolitan workhouse schools.

### Our Needlework Competition.

CHRISTMAS, 1895.

ONCE again our readers have responded to our appeal for gifts for patients who spend Christmas Day in hospital, and we have gladly received and distributed the contents of the parcels sent to us. A number of the articles were marked "Not for competition," and some nice parcels did not contain even the names of the donors, but were none the less welcome.

We are much impressed by the fact of almost every offering having come from nurses, and knowing as we do how very little time they have to spare, we value very highly all work done by them. We cannot but regret that so little help has come from those whose leisure and means so far exceed those of always-busy nurses. Convalescent private patients and their friends might surely remember the existence of certain other invalids to whom recovery from illness means an immediate return to hard work, scanty meals, and insufficient clothing. A few warm garments are of inestimable value to hospital inmates who leave so many comforts behind them when they exchange the ward surroundings for those of their own homes.

The task of distribution, generally one of our pleasantest annual experiences, has been rather overshadowed this year by the inadequacy of the supply to meet the demands from many quarters. "We have never been so badly off for Christmas presents for the patients" has been a constant remark to us during the last week. "No one has sent anything for the men; we want socks and shirts so badly."

One or two people have seasoned their lamentations with suggestions that the noble sums which have been given this year to the hospitals and the Pension Fund may have naturally curtailed small individual contributions. However, this is hardly a feasible explanation, as the big subscriptions come from quite other sources. If the women, who are so often heard regretting the cir cumstances which have kept them from adopting the calling of a nurse, would only realise that they could do equally useful and necessary work for the sick outside the ward, they would benefit themselves as well as clothe the patients.

We have always advocated the union of outside visitors with the regular hospital workers, for whilst the latter are actually in the service of the sick the former can supplement their labours in many useful ways. If every reader of THE HOSPITAL would decide to send one garment each year for our Christmas distribution we should never again have an insufficient supply.

Our prizes this year have been awarded as follows: Miss Constance Delap 10s. for a pretty bed jacket, which must have taken much time to finish so daintily; Nurse Clark 10s. for a beautifully-made flannel shirt of an excellent shape, which was accompanied by a second equally good one not for competition; Nurse Reeve 5s. for a particularly soft and warm petticoat; Nurse Aston 5s. for a very pretty over-petticoat.

The socks, not so numerous as usual, still presented a good variety of knitting, several being very well done. Those for which the first prize of 5s. was allotted to Nurse E. Elliot were beautifully shaped, and she kindly sent us two other pairs. The second prize, 2s. 6d., was given to Nurse Mackie, whose socks were very well shaped and finished; Nurse Bishop received 3s. for a knitted vest. Nurse Agnes Gouldsmith sent a parcel modestly described by herself as containing "one or two articles for Christmas gifts to the inmates of hospitals." Policy Holder 370 and 1,240 also sent a nice parcel of useful things, and a friendly note explaining that not having been able to work for us this year as she has formerly done, she made some purchases at the Trained Nurses' Club Sale of Work. Nurse Prior sent a warm petticoat and some stockings; Miss Delay, a scarlet flannel petticoat; Nurse Bishop, a petticoat and waistcoat; Policy Holder 37, a charming flannelette night-dress; Miss Lockyer, shawls; Nurse Willson and Miss E. Clarke, parcels of warm articles; and two other parcels sent anonymously contained two capital petticoats, socks, vests, and jerseys. Socks came from Nurses Chapman, Alice Wood, Garriock, Dunn, Baker, Bartlett, L. Lowson, Jackson, Snell, Griffiths, Boyd, Salt, and Miss Richardson. Nurse Newsome has sent an acceptable present of eight vests, and from Miss Pritchard we have also received seven delightful vests and some stockings. Nurse Emily Elliot has made a baby's jacket and a beautifully-shaped pair of gaiters. Madame Monchablon's parcel contained some beautiful needlework, bed jackets, petticoats, and flannelette night gowns, besides many other useful articles, such as cuffs, scarf, and frocks. Miss White sent three famous scrap books.

We have to acknowledge the kind interest and assistance of Miss H. Gordon, Miss Robinson, and Miss Lamport, for, as our readers will remember in previous years, we always secure independent judges of our contributors' needlework. Parcels have been sent to the Great Northern Hospital, the London Hospital, Charing Cross Hospital, Royal Ophthalmic Hospital, St. Thomas's Hospital, Middlesex Hospital, Metropolitan (Kingsland Road), King's College Hospital, University Hospital, Hospital for Women (Waterloo Bridge Road), Mercy Hospital, co Galway, and East-end Mothers' Home.

### Novelties for Nurses.

BASIN SYRINGE CLIP.

Messrs. Reynolds and Branson never allow us to forget their special gift for useful innovations. The last novelty they have brought to our notice is a little clip to prevent the syringe slipping away from the basin when in use, a matter that is difficult to prevent without some such arrangement. This inexpensive and simple little contrivance will be found invaluable for those who possess an ordinary syringe, such as is depicted in our illustration. When the enema has been used, the nozzle is to be inserted in the ring for the purpose, and thus allowed to drain into the basin without immersion in the liquid. This latter convenience renders the clip of value even where the syringe with indiarubber suction end is in use, and therefore it should be universally adopted. We recommend our readers to write for one of these useful little clips to the manufacturers, at 13, Briggate, Leeds, or to order one through their chemist.

### Everybody's Opinion.

[Correspondence on all subjects is invited, but we cannot in any way be responsible for the opinions expressed by our correspondents. No communications can be entertained if the name and address of the correspondent is not given, or unless one side of the paper only be written on.]

### REAL OR SHAM NURSES.

"Policy Holder 47" writes: Let those nurses who are anxious to be known as the "real" ones accept the suggestion made by "Policy Holder 1,399." Outdoor uniform is not all important, and I can understand it being of much greater value when worn by the pioneer workers, whose love carried them into the midst of wretchedness and depravity, such as the majority rarely enter into. I fail to see how members of the R.N.P.F. can represent themselves to the general public as the only real nurses. That would be indeed hard on those who, from no fault of their own, are unable to join the Fund. Again, how specially confusing it would be in institutions where some nurses are not policy holders. As to submitting the matter to our gracious President, I think it would be better to claim her help for a more momentous cause, and not trifle with the great kindness already shown by "Our Princess."

"Policy No. 3,162" writes: I should like, if possible, to help the nurses who are making suggestions to prevent the abuse of uniform. I fail to see the consistency of the suggestion made by "A Lover of a Bonnet and Cloak," as many institutions prefer their own particular bonnet and cloak. I think a badge, designed, patented, and made copyright, to be worn outside the cloak would be more to the point. The armlet is very nice indeed, but that unfortunately cannot be worn on a cloak. I hope some of the Pension Fund nurses will make more suggestions concerning a badge, and "our Princess," if petitioned, would probably help us."

### MALE NURSES.

"Nurse Marie" writes: This summer I was for a few months in New York, in one of the hospitals, where they had male probationers for the male wards. They were trained in every respect like the female nurses, and the plan seemed to answer admirably. The men appeared in every way fitted for their work, and in cases of delirium and night accidents (when the patients were often under the influence of drink), one realised the comfort derived from the presence of a trained male assistant. Just lately a nurse has been summarily dismissed from a nursing home for refusing to go to a certain male patient. I could not learn that the case was in any way fitted for any woman to attend, and most certainly not for a young woman. This is only one case of many, and I think they all show the desirability for the introduction of trained male nurses. Why (as you say in The Hospital) should the systematic instruction of men be practicable in New York and not over here?

### First Aid at Aldershot.

His Royal Highness the Duke of Connaught distributed the first aid certificates to a number of members of the St. John Ambulance Association at Aldershot last week. The distribution, which took place in the Army Gymnasium, was preceded by a display of first aid by the Artillery, Engineers, Military Police, and Fire Brigade. The Duke gave an interesting little address, and spoke of having had a very satisfactory proof in his own household of the practical value of the St. John Ambulance teaching, for his carpenter, who had been in the Scots Guards and had gone through the course, had successfully arrested hæmorrhage in the case of a man who had cut his femoral artery.

### St. Catherine's Home, Bradford.

### ANNUAL MEETING.

An excellent record of work was laid before the annual meeting held in connection with St. Catherine's Home, Bradford. The chair was taken by the Ven. Archdeacon Bardsley, and the report was read by Dr. Rabagliati for Mrs. Rabagliati, the indefatigable hon. secretary. Additional contributions are required to enable the ward furnished by Mr. Illingworth to be utilised. The patients in the home are all suffering from incurable diseases, chiefly cancer or advanced phthisis, and generally "come to stay" when they enter the pleasant house which generous friends have provided for them. The management of this little institution is exceptionally good and remarkably economical, and the matron, Miss Hadley Scott, is to be congratulated upon her able administration. Many kind friends display unwearied interest in the patients, and bring much brightness and comfort to them, and in all ways forward the welfare of all connected with the deserving charity.

### A Tribute to Medical Women.

By a majority of votes the Chelsea Guardians have decided to advertise for a second assistant medical officer for their infirmary, stating that women doctors will be eligible as candidates for the post. Several Guardians remarked that they had been informed of the devotion to their duties which medical women had exhibited in other institutions. Attention was drawn to the fact that there were 800 women and 270 girls, and that they might be advantageously treated by a doctor of their own sex.

### Minor Appointments.

Indian Nursing Service.—Miss Alice M. Rackham has been made a Sister in the Indian Nursing Service. She was trained at the Norfolk and Norwich Hospital, Norwich, and at Guy's Hospital, and held the post of Staff Nurse at the Lincoln County Hospital. We wish her success in her new work.

### Where to Go.

At the "Dreadnought" Seamen's Hospital, Greenwich: On Christmas Day morning service was held in the chapel at half-past ten. A dinner of turkey and pudding was served to the patients, and an entertainment arranged in the evening. On January 3rd there will be a Christmas tree for the children who have been in the hospital, and on January 7th an entertainment for the nurses.

### Notes and Queries.

### Queries.

(62) *Publisher.*—Who is the publisher of Dr. Keith's "Plea for a Simple Life" noticed in The Hospital last week?—*Reader.*

(63) *Probationer.*—Please let me know at once why the advertisement sent last week was not inserted.—*Liverpool.*

(64) *Abroad.*—Can you tell me of any institution which sends out district nurses and pays their passage to India, New Zealand, or Australia?—*H. N.*

(65) *Invalid's Chair.*—Will you tell me where I can get the wheels (with rubber) for an invalid chair?—*M. A. T.*

### Answers.

(62) *Publisher (Reader).*—Dr. Keith's book is published by Messrs. Adam and Charles Black, Soho Square, London.

(63) *Probationer (Liverpool).*—Your advertisement is not inserted because the wording is unsuitable. We wrote and explained this, sending our answer to the only address you had given us. Our letter was returned endorsed at the Post Office, "Fictitious name, contrary to regulations." If you send us your address we will return the stamps which you enclosed.

(64) *Abroad (H. N.).*—We have not heard of any institution of the kind. District nurses if well trained, can get plenty of work in England, and are sorely needed in many places.

(65) *Invalid's Chair (M.A.T.).*—From Messrs. Alfred Carter, 47, Holborn Viaduct, E.C., or Messrs. Leveson and Sons, 90 and 92, New Oxford Street, W.C.

### The Half-Trained Fever Nurse.

By a Medical Superintendent.

### A SCHEME FOR TRAINING HER.

Undue specialism is objectionable, and the formation of a Sanitary Nurses Association is only justified by a conviction that the fever-nurse has come to stay. If, on the one hand, the managers of the fever hospitals who, on the ground of economy, now employ probationers, could be persuaded to engage for the future only competent nurses possessing a general certificate; and on the other, it could be guaranteed that a sufficient number of good-class applicants would be forthcoming to fill the vacancies: then the unfortunate necessity for evolving a special genus of nurse would disappear. As it is, however, it is better to face the necessity, and, as far as may be possible, to make the difference between the fever and the general nurse one of kind rather than class —of work rather than status.

It is proposed, then, to formulate a scheme for the organisation of fever nurses, keeping in mind that the main problem is the suppression of the half-trained nurse. As bearing on this problem the scheme is of vital significance to the general nurse; but I would ask the fever-nurse to remember that it is of still greater importance to her as a suggestion framed in her interests, the acceptance or rejection of which is in her own hands. Let me say here that the whole blame for the fever-nurse being half-trained must fall upon the institutions which engage her as probationer. The managers of such institutions no doubt find her economical. They do not, however, seem to realise that they owe her something in the place of pounds, shillings, and pence. It is impossible to feel anything but sympathy for the well-educated woman who becomes a fever-probationer under such conditions; as for the ill-educated probationer, the sooner she becomes extinct the better.

The proposed Sanitary Nurses' Association would strive, by means of circulars and pamphlets addressed to fever hospital committees, medical officers of health, resident and visiting physicians, and matrons—and also through the influence of members locally exerted—to do away with the half-trained fever nurse. In order to gain this end it would urge the adoption of a uniform two or three years' course of practical and theoretical training, the latter to include (1) elementary anatomy and physiology; (2) dispensing; (3) hygiene; (4) outlines of general medical and surgical nursing; (5) in special detail—the pathology, clinical history, management, nursing, and treatment of the various fevers. It will be apparent that only hospitals of a certain size, possessing special facilities for the training of probationers, could undertake such a curriculum. It would be for the Association to say whether the certificate granted by any given institution should or should not entitle its possessor to become a member. That it would be to the interest of hospitals fitted for the purpose to adopt the course of training recognised by the Association is obvious, since it would enable them to obtain the best class of probationer at a minimum salary; indeed, I have little doubt that in time a premium would be forthcoming. In justice to the competent nurse who might have already taken up fever work, membership at the outset might depend on special conditions calculated to exclude those falling below a certain standard as regards education and training. After a reasonable time, however, only fever nurses who had undergone the course of training indicated above would be admissible as members, who would wear a special badge, and adopt the title of "sanitary nurse"—the designation "fever nurse" being objectionable on the same grounds as those which led Dr. Thorne, in his "Report on Isolation Hospitals" (page 33) to recommend the disuse of the term "fever hospital"; "sanatorium" and "sanitary hospital" having come into vogue, "sanitary nurse" follows as a logical consequence. The admission of general nurses taking up fever work after the constitution of the Association could be made dependent on their working for one year as assistant or charge nurse in a recognised fever hospital. The sanitary nurse, in or out of hospital, would be expected to adhere solely to fever work; but a general certificate would do away with this restriction. It may seem that it is giving undue prominence to a minor point, but signs are not wanting that it would be well to adopt as a fundamental principle of the association the subservience of the nurse to the physician. Finally, an effort would be made to enlighten by all legitimate means, however drastic, those local authorities whose notions of the construction and management of fever hospitals belong to the middle ages. The council of the association would consist of medical officers of health, resident and visiting physicians, matrons, and sisters, with an executive committee meeting in London. Success would greatly depend on a reasonable subscription—say half-a-crown per annum.

It may seem at first sight that an association of the kind described would have no real power, but when it is to the interest of all concerned that certain things should be brought about, approval, if not active support, may be counted upon. The association would benefit. (1) The fever-trained nurse, by raising her to a position differing in kind, but not in degree, from that enjoyed by the general nurse. (2) The general nurse, by removing one source of the half-trained nurse and so improving the status of the profession as a whole; and also by suppressing to some extent unfair competition on the part of nurses with doubtful qualifications—competition which, in the absence of stringent measures to put it down, promises soon to assume disastrous proportions. (3) To the public, which has no guarantee that a nurse is trained beyond the fact that she wears a uniform. (4) To the managers of fever hospitals for reasons already stated. (5) To the patient, whose welfare must ever be the fundamental consideration.

For the rest, publicity is a powerful agent when fairly employed, and the association would, no doubt, soon have behind it a sufficient force to carry out its programme.

### Royal British Nurses' Association.

The second sessional lecture of the season on "Mountain Myths and Legends," by Mr. Clinton Dent, will be delivered on Friday, January 31st, instead of January 24th, as previously announced.

The quarterly meeting of the General Council will be held on Friday, January 10th, 1896, at five p.m., at 17, Old Cavendish Street.

Some response has been elicited from members to a suggestion made in the November number of the *Nurses' Journal* (page 83) by Miss E. J. M. Mackenzie as to a practical method of assisting the finances of the association. The names of all these members and friends will be published in the February issue of the journal.

### Presentation.

On the resignation of Miss Cooke, matron of the Dreadnought Seamen's Hospital, Greenwich, the past and present officers and nurses of the "Dreadnought" have presented her with a testimonial in the form of a purse of considerable value.

On leaving Brigg, Miss A. Leigh was presented with a handsome case of silver-mounted ebony-back brushes by the members of the Ladies' Committee as a token of their appreciation of her work as District Nurse.

### The Princess Maud Marriage Present Fund.

The list of subscriptions to this fund will close on the 31st inst. The rest of the names will be inserted in our next issue, and the total amount received will then be noted.

## Figures and Tables

**Figure f1:**